# Fungal Elicitor MoHrip2 Induces Disease Resistance in Rice Leaves, Triggering Stress-Related Pathways

**DOI:** 10.1371/journal.pone.0158112

**Published:** 2016-06-27

**Authors:** Najeeb Ullah Khan, Mengjie Liu, Xiufen Yang, Dewen Qiu

**Affiliations:** 1 The State Key Laboratory for Biology of Plant Diseases and Insect Pests, Institute of Plant Protection, Chinese Academy of Agriculture Sciences (CAAS), Beijing, 100081, China; 2 Institute of Biotechnology and Genetic Engineering, The University of Agriculture Peshawar, Pakistan; Nanjing Agricultural University, CHINA

## Abstract

MoHrip2 *Magnaporthe oryzae* hypersensitive protein 2 is an elicitor protein of rice blast fungus *M*. *oryzae*. Rice seedlings treated with MoHrip2 have shown an induced resistance to rice blast. To elucidate the mechanism underlying this MoHrip2 elicitation in rice, we used differential-display 2-D gel electrophoresis and qRT-PCR to assess the differential expression among the total proteins extracted from rice leaves at 24 h after treatment with MoHrip2 and buffer as a control. Among ~1000 protein spots detected on each gel, 10 proteins were newly induced, 4 were up-regulated, and 3 were down-regulated in MoHrip2-treated samples compared with the buffer control. Seventeen differentially expressed proteins were detected using MS/MS analysis and categorized into six groups according to their putative function: defense-related transcriptional factors, signal transduction-related proteins, reactive oxygen species (ROS) production, programmed cell death (PCD), defense-related proteins, and photosynthesis and energy-related proteins. The qPCR results (relative expression level of genes) further supported the differential expression of proteins in MoHrip2-treated rice leaves identified with 2D-gel, suggesting that MoHrip2 triggers an early defense response in rice leaves via stress-related pathways, and the results provide evidence for elicitor-induced resistance at the protein level.

## Introduction

Plants are exposed to various groups of pathogenic microorganisms at every stage of growth and have evolved effective defense mechanisms to cope with the encountered microorganisms. They have the ability to distinguish pathogen-derived compounds known as elicitors, mostly composed of lipids, proteins or glycoproteins [1]. Elicitors of the pathogen trigger plant defense responses such as a burst of reactive oxygen species (ROS), which results in a rapid and highly programmed cell death (PCD) [2], also known as the hypersensitive response (HR) [3], and the generation of nitric oxide (NO), all of which are considered as a signal of innate immunity in plants and the source of resistance to biotic and abiotic stresses [4–6]. Ultimately, elicitors of the pathogen activate plant basal responses, such as the biosynthesis of antimicrobial secondary metabolites (e.g. phytoalexins) and induce the expression of defense-related proteins including pathogenesis-related proteins (PR) [4,7,8]. Consequently, elucidating the mechanism underlying elicitor-induced plant responses is important for understanding plant–pathogen interactions and developing new disease control strategies.

Grain yield of rice (*Oryza sativa*) can be greatly decreased by abiotic and biotic stresses, including rice blast caused by the fungus *Magnaporthe oryzae*. The availability of rice genome and proteome databases has now enabled studies in the field of rice proteomics and biological processes including responses to abiotic and biotic stresses [9]. Differentially expressed proteins have been identified in rice leaves and suspension cells treated with blast fungus (*Magnaporthe oryzae*) and bacteria (*Xanthomonas oryzae*) [10,11]. Various proteins, including PR proteins (PR5 and PR10) are induced in rice leaves treated with chitosan and fungal or bacterial preparations [12]. Rice cells cultured in suspension containing crude extract of the blast fungus are induced to synthesize PR5, PR10 and the induced proteins that are also induced by salt stress [13]. Several studies have been conducted on elicitor-treated rice seedlings and identified proteins including ROS, PCD, signal transduction and energy-related proteins that are differentially expressed in fungal elicitor-treated rice leaves compared with controls [14,15].

Recently, various types of elicitors have been used to improve plant resistance to pathogens [16–18] by activating different types of defense responses such as signaling pathways, ROS, HR response, NO production, and PCD [19]. MoHrip2, an elicitor protein of the rice blast fungus (*Magnaporthe oryzae*), can induce HR and the production of NO and hydrogen peroxide (H_2_O_2_) in tobacco leaves. Moreover, rice seedlings treated with MoHrip2 have shown an induced resistance response to conidia of *M*. *oryzae* [18]. Defense-related genes, including transcription factors (TFs), salicylic acid (SA), jasmonic acid (JA), and PR genes were induced within 24 h (hpt), also SA and JA synthesis was up-regulated in MoHrip2-treated rice seedlings indicating that MoHrip2 induced disease resistance in rice, while activating various stress-related pathways genes (Unpublished data). However, little is known about the mechanism of this induced resistance, which may provide another strategy for controlling rice blast. To begin to elucidate the molecular mechanism underlying the induction of resistance in rice plants by Mohrip2, analyzing proteins via differential-display 2-dimensional polyacrylamide gel electrophoresis (2D-PAGE) is therefore crucial.

In the present study, rice cultivar Nipponbare (*Oryza sativa* spp. *japonica*) seedlings were treated with the elicitor protein MoHrip2, and then the proteins were analyzed using differential-display 2D-PAGE and tandem mass spectrometry (MS/MS). The selected genes that encode the differential-display proteins were also analyzed by qRT-PCR.

## 2. Materials and Methods

### 2.1. Elicitor MoHrip2 Preparation

**Fig 1:**
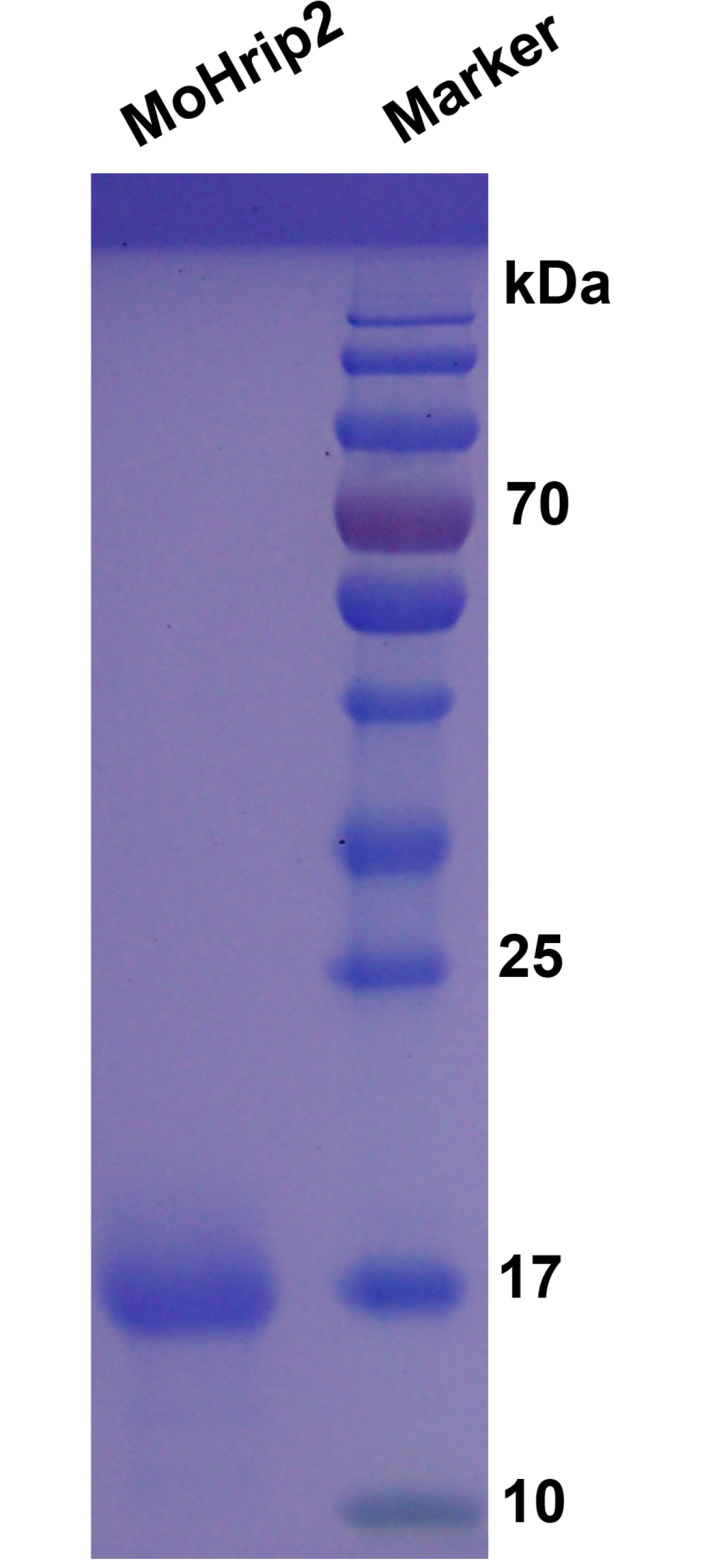
Confirmation of purified MoHrip2 with SDS-PAGE. The purified MoHrip2 was run on SDS-PAGE, stained with Coomassie brilliant blue, and shown to be 16 kDa in size compared with the marker (PageRuler Pierce 26616, Thermo Scientific).

The recombinant MoHrip2 protein elicitor (NCBI accession AFK29792) was purified as described before [18]. Briefly, *E*. *coli* (containing the MoHrip2 gene) was first grown in 50 mL LB broth for 5 to 6 h at 37°C, then 10 mL of this LB broth containing *E*. *coli* was transferred to 1 L fresh LB broth for 3 to 4 h of culture at 37°C. Synthesis of the elicitor protein was induced, adding 0.2 mM IPTG (isopropyl β-d-1-thiogalactopyranoside) to the culture, which was then grown overnight at 16°C. MoHrip2 was isolated from 1 L LB broth with a detergent washing method [20]. The MoHrip2 protein was purified on a His-Tag column (TransGen Biotech, Beijing, China) and desalted on a Millipore column (Merck, Germany). The purified MoHrip2 was quantified with a Bradford assay and confirmed by SDS-PAGE “Fig 1”.

### 2.2. Rice Growth Conditions and Samples Preparation

Sixteen rice (*Oryza sativa* spp. *japonica* cv. Nipponbare) plants per pot were grown as described before [20] to the three-leaf stage in a growth chamber at 28 ± 2°C, 75 ± 5% relative humidity, and 14 h light/10 h dark [21]. The plants were sprayed either with MoHrip2 (800 μg/mL of buffer) or with the buffer (50 mM Tris HCl) as a control. After 24 h, one sample (0.5 g leaves) was collected from each of three biological replicates to extract total proteins, and 100 mg leaf tissue was similarly collected after 0, 12, 24, and 48 h to extract total RNA. All samples were frozen in liquid nitrogen and stored at −80°C until use.

### 2.3. Total Protein Extraction

The leaf sample (0.5 g) was ground to a powder in liquid nitrogen using a mortar and pestle and then dissolved in pre-chilled 10% (w/v) trichloroacetic acid (TCA) and 0.07% (v/v) 2-mercapto-ethanol (2ME) in acetone, mixed well and incubated overnight at −20°C. After centrifugation (16,000 × *g*, 20 m at 4°C), the pellet was washed and centrifuged (16000 × *g*, 20 min at 4°C) three times with pre-chilled 80% (v/v) acetone. The pellet were dissolved with 1.5 mL lysis buffer (7 M urea, 2 M thio urea, 4% CHAPS, 20 mM Tris HCl, pH 8.5) and incubated overnight at room temperature with gentle shaking. After centrifugation (18,000 × *g*, 1 h at 4°C), the supernatant was transferred to a new tube, the pH was adjusted to 8.5, and the protein concentration was measured using the Bradford method [22]. The crude protein was stored at −80°C until use.

### 2.4. 2D-PAGE

The 2D-PAGE was done using a standard protocol [23]. The protein sample (1000 μg/mL) was purified using a 2-D gel clean up kit (GE Healthcare) according to the manufacturer’s instructions, and subsequently dissolved in 450 μL loading buffer (8 M urea, 2% w/v CHAPS, 0.5% v/v IPG buffer, 40 mM dithiothreitol (DDT), and 0.002% bromophenol blue). Isoelectric focusing (IEF) was performed at (30 V for 6 h, 60 V for 6 h, 200 V for 1 h, 500 V for 1 h, 1000 V for 1 h, 8000 V for 2.30 h, 8000 V for 7 h, and 500 V for 5–20 h), at 20°C for a total of 65,000–70,000 VH, using linear 24 cm strips (pH 4–7) in an IPGphore system (GE Healthcare). Vertical SDS-PAGE (12%) was run at a constant wattage (2 W per strip for 45 m, 9 W per strip for 10 h) and was stained with Coomassie blue G-250 (Thermo Scientific) according to the manufacturer’s instructions.

### 2.5. Gel Scanning and Differential Display Protein Analysis

The gels were scanned using an image scanner II (GE Healthcare) at 300 dpi resolution, and the images were saved as TIF. The images were then analyzed with Image Master 5.0 software (GE Healthcare). Spots were detected, measured and the background was subtracted according to the software instructions (GE Healthcare). The spots were matched using an automatic method and were carefully edited using the software 5.0 users instructions (GE Healthcare). Subsequently, two groups were created as the control and treated gels of the corresponding three biological replicates. Spot volumes were quantitatively analyzed. The differential-display proteins were identified based on the criteria that (i) the differential spots were reproduced in three replicates and (ii) spot volumes differed at least two-folds.

### 2.6. MS/MS Detection and Database Search

Protein spots were cut from the gel, in-gel digested with trypsin, and protein was extracted as described previously [24]. Briefly, C18-ZipTips (Millipore Billerica MA, USA) were used for MALDI target preparation according to the manufacturer’s instructions. The peptides were desalted, concentrated and eluted onto the MALDI target using α-cyano-4-hydroxycinnamic acid (CHCA) solution (100 pmol/μL). The peptide mass fingerprint (PMF) was analyzed for each sample and processed using DataExplore software (Bruker Daltonics, Bremen, Germany) provided with the equipment. The data were analyzed using the Mascot (http://proteometrics.com/protocol-cgi/profound.exe). The following mass spectrometry parameters were selected for the database search: the spectra were recorded in a mass range of 800 to 4000 Da; the error range of the apparent isoelectric point was 60.5 pH; the error range of the apparent molecular weight was 620%; fixed cysteine modification with carbamidomethyl; samples were derived from *Oryza*; allowed nonrestriction sites was 1; minimum matching peptide fragments was 5; trypsin fragments were run with a mass tolerance of 200 ppm; mass values were MH+. The proteins were identified based on the search results and the Ip and MW values in the gel.

### 2.7. Total RNA Isolation, cDNA Synthesis, and qRT-PCR

Total RNA was extracted from control and treated rice samples using an EasyPure Plant RNA Extraction Kit (TransGen Biotech, Beijing, China), according to the manufacturer’s instructions. The concentration of RNA was measured using an Eppendorf BioPhotometer plus. Approximately, 500 ng RNA for each sample was reverse transcribed using All-in-One First-Strand cDNA Synthesis Super Mix for qPCR (TransGen Biotech). Primers were designed using the online NCBI primer blast software according to the gene sequences in the GeneBank database “Table 1”. *OsActin* was used as an internal control [25]. The qRT-PCR was performed in a Bio-Rad iCycler iQ5 using TransStart Green qPCR Mix UDG (TransGen Biotech), according to the manufacturer’s instructions. In brief, each 20 μL reaction volume contained 2 μL cDNA, 10 μL qPCR mix, 0.5 μL of each primer (10 mM) and 7 μL ddH_2_O. The following thermal conditions were used: initial denaturation at 94°C for 5 min; 35 cycles of denaturation (20 s at 94°C), annealing (30 s at 60°C) and extension (30 s at 72°C). The melt curves were run immediately after the last PCR cycle to measure the influence of induction. The qPCR was performed in three biological replicates. Induction of gene expression was assessed using delta–delta method [26]. The results were analyzed using the One-way ANOVA with Tukey post test using GraphPad Prism version 5.00 for Windows, GraphPad Software, San Diego California USA, www.graphpad.com.

**Table 1:** Primers used for qRT-PCR analyses.

Name	Forward primer	Reverse primer	Accession
*OsActin*	CGACTGGAACTCGCTCATCA	ACACCAACAATCCCAAACAGAG	AK060893
*OsGLP*	TGCCCAATCAGCACTCAGTA	TTTCCACCTGAAACGCCTTG	BAT04433
*OsTLP* (*PR 5*)	TCACCTGCAGGGACAGC	GGGCAGAAGACGACTTGGTA	BAT18199
*OsPR 10*	TCAACCCTGCTGTGGATGAT	CTTGAGCTTGCCCACCTTAC	BAT17596
DnaK	CTTGGAAAGCTGAGGAGGGA	TCTTGACAGGTCCCATGGTC	BAS86012

## 3. Results

### 3.1. Differential-Display Proteins after Treatment with MoHrip2

**Fig 2:**
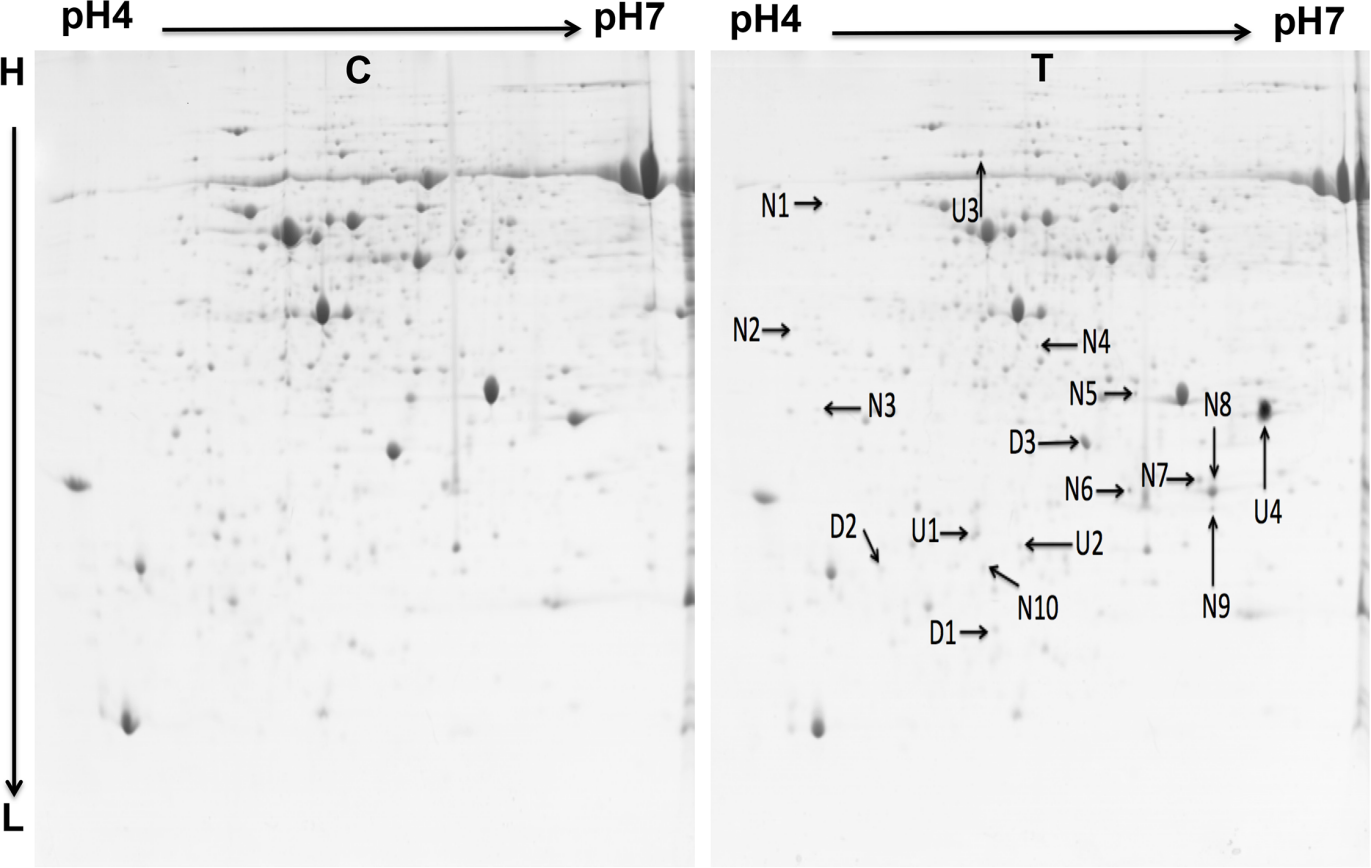
Two representative 2D-gels of control (C) and MoHrip2-treated (T) rice leaves at 24 h after treatment. Representative 2-DE patterns of proteins from rice leaves (2^nd^ and 3^rd^ leaves) inoculated with 50 mM Tris HCl as a control (C) or the elicitor MoHrip2 (800 μg/mL) (T). Total extracted proteins (1000 μg) were loaded on 24 cm IPG strip (GE Healthcare) with a linear gradient of pH 4–7 for IEF, following electrophoresis of 12% SDS-PAGE and Coomassie (G-250) staining. Arrows on the gel indicate the positions of newly induced (N1–N10), up-regulated (U1–U4) and down-regulated (D1–D3) proteins compared with proteins in the control. The H and L on the left indicate separation of proteins from high to low molecular weight.

**Fig 3:**
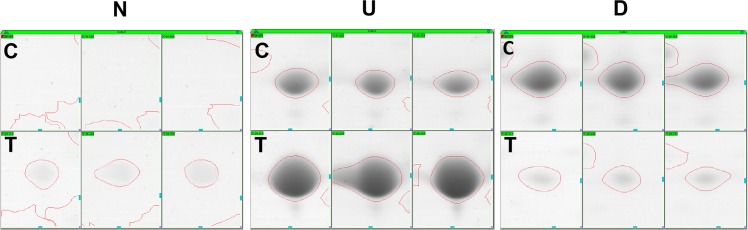
Proteins spots in a matched set representing proteins newly induced (N), up-regulated (U) and down-regulated (D) 24 h after treatment of rice with MoHrip2. Close-up views of the proteins spots of the 2-DE gels that show significant differences in protein expression between controls (C) and treated (T) rice leaves. Proteins (1000 μg) were loaded on 24 cm IPG strip with a linear gradient of pH 4–7 for IEF, following electrophoresis of 12% SDS-PAGE and Coomassie staining for data analysis. Spots were detected using software 7.0 (GE Healthcare) in three replicated gels.

**Fig 4:**
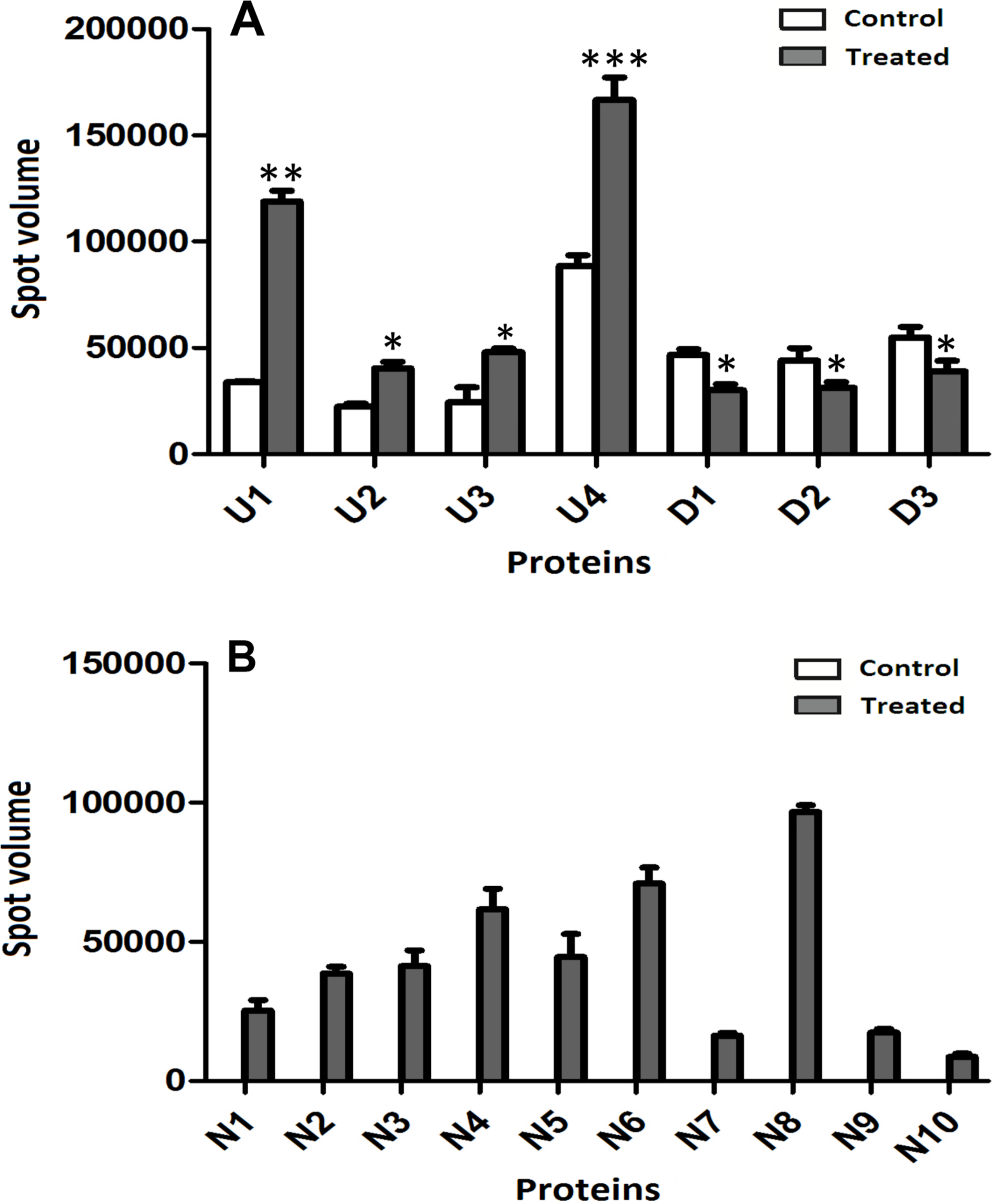
Relative levels of proteins differentially expressed in rice leaves at 24 h after treatment with the elicitor MoHrp2. Changes in protein spots were calculated using Image-Master 5.0 software (GE Healthcare) and plotted as the relative spot volume of 17 spots indicated in Fig 2. Values are the actual volumes of the proteins spots from three independent experiments. Unshaded (control) and shaded bars (treated) represent variation in spots volume, up- (U) and downregulated (D) spots (Fig 4A) and newly induced (N) proteins (Fig 4B). Error bars indicate ±SEM; essentially identical results were obtained for each spot in three independent experiments. The asterisks *, ** and *** indicate significant difference at (*p* = 0.05, *p* = 0.01 and *p* = 0.001, t-test), respectively as compared to controls (4A). The newly induced proteins (4B) are significant as the control values are zero.

About 1000 protein spots were stained with Coomassie brilliant blue G-250 and detected using Image Master 5.0 software (GE Healthcare) on each gel for MoHrip2-treated or buffer-treated samples. Two representative gels “Fig 2” and one for each differential-display protein spot for newly induced, up- and down-regulated are shown “Fig 3”. Both gels are of high quality and suited for analyzing the differentially expressed proteins. Using the differential-display criteria, expression changes in 17 proteins were detected using Image Master 5.0 software (GE Healthcare) at 24 hpt: 10 (N1–N10) were newly induced “Fig 4A”, four (U1–U4) were up-regulated and three (D1–D3) were down-regulated “Fig 4B”.

### 3.2. Identification of Differential-Display Proteins by MS/MS Analysis

The differential-display proteins from the gels with MoHrip2-treated proteins were excised and analyzed by MS/MS. These 17 differential-display proteins “Table 2”, with the exception of two uncharacterized proteins (N1 and N3), were allotted to six groups according to their putative function: (i) defense-related transcriptional factor (N2), (ii) signal transduction-related proteins (N4, N7, N9), (iii) reactive oxygen species (ROS) (N5, D1, D2, D3), (iv) programmed cell death (PCD) (N6), (v) defense-related proteins (N10 and U4), and (vi) photosynthesis and energy-related proteins (N8, U1, U2, U3).

**Table 2:** MS/MS identification of differentially expressed proteins, newly induced (N1–N10), up-regulated (U1–U4) and down-regulated (D1–D3), from rice leaves 24 h after treatment with the elicitor MoHrip2.

Spot	Protein name	Accession	Protein score	Theoretical pI/MW (kDa)
N1	Putative uncharacterized protein	BAS76295	30	7.10/42.10
N2	Zinc finger protein	BAS74060	23	5.50/35.33
N3	Putative uncharacterized protein	BAS95372	34	5.68/33.07
N4	Tricin synthase 1	BAT06083	328	5.10/27.80
N5	Germin like protein	BAT04433	182	5.48/24.60
N6	Putative SAG12 protein	BAF04841	42	6.44/39.44
N7	Putative SP3D	BAS71052	42	8.89/29.83
N8	4-Coumaroyl-CoA synthase 5	BAT05682	32	5.81/57.00
N9	Putative farnesylated protein	BAT05353	25	9.68/16.71
N10	Thaumatin like protein	BAT18199	77	5.07/17.98
U1	RuBisCO large chain	CAG34174	172	6.22/52.80
U2	Magnesium ion binding protein	BAS74724	131	6.35/28.80
U3	Magnesium ion binding protein	BAT16303	154	9.10/58.99
U4	Pathogenesis-related protein 10	BAT17596	448	4.88/16.90
D1	Peptidyl-prolyl *cis-trans* isomerase	BAS89404	48	5.77/57.10
D2	Protein disulfide isomerase	BAT13067	604	5.01/56.90
D3	DnaK protein	BAS86012	325	5.09/73.34

### 3.3. qRT-PCR Analysis of Differential-Display Proteins

**Fig 5:**
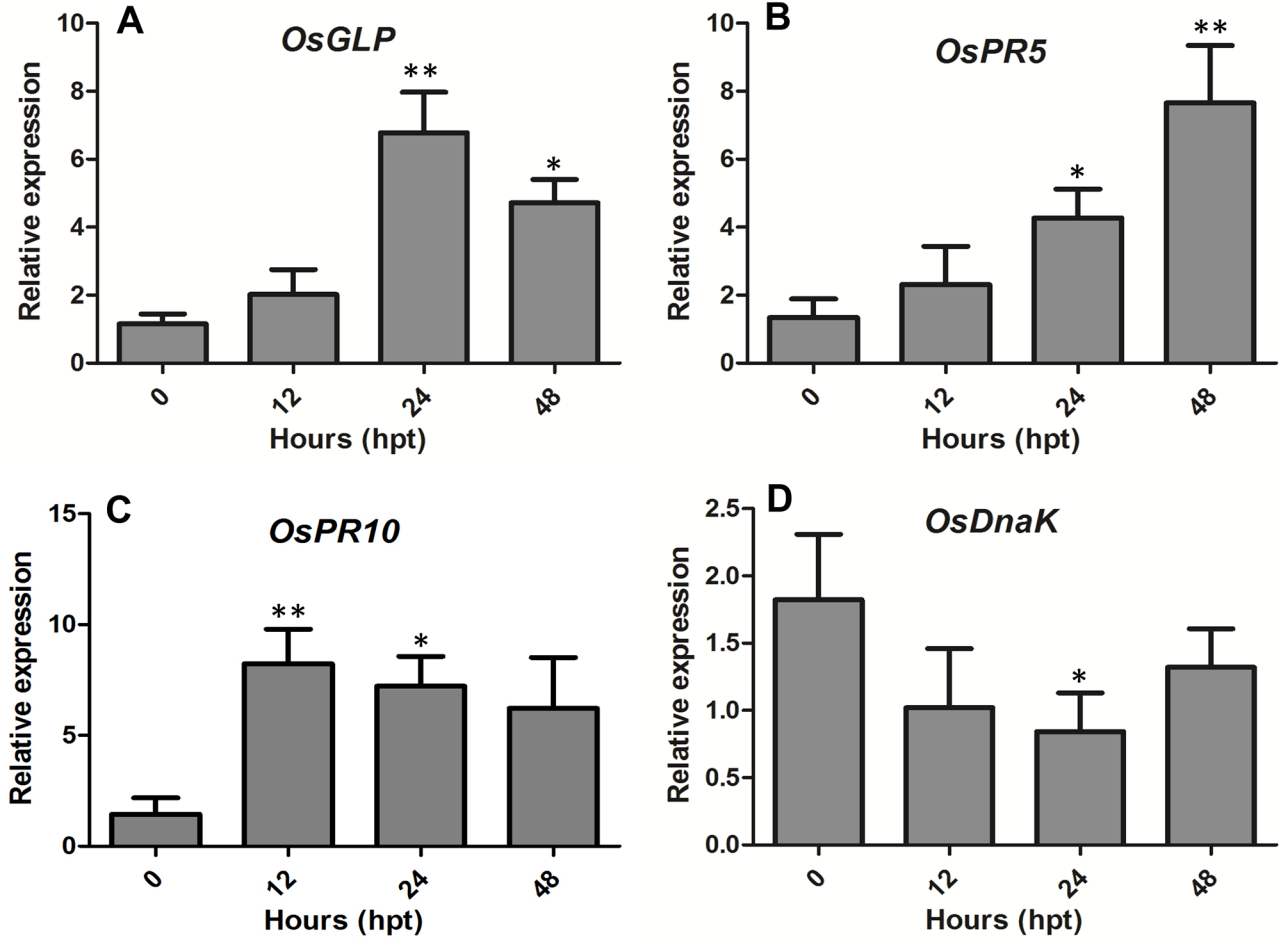
Relative expression level of genes encoding differentially expressed proteins in rice leaves at various times after treatment with MoHrip2. Relative expression (±SEM) is the fold-change at the given hours post treatment (hpt) compared with the control. Relative expression was analyzed by using quantitative RT-PCR, and expression levels were normalized to those of the actin gene as a housekeeping gene. *OsGLP* (5A) and *OsPR5* (5B) represent newly induced, *OsPR10* (5C) upregulated and *OsDnK* (5D) represent downregulated proteins. Error bars indicate ±SEM; essentially identical results were obtained for each gene in three independent experiments. The asterisks * and ** indicate significance (*p* = 0.05 and *p* = 0.01, ANOVA), respectively.

Four proteins, two newly induced (N5 and N10) and one up-regulated (U4) and one down-regulated (D3), were analyzed with qRT-PCR over time (0–48 h after treatment, hpt). Expression levels of genes encoding the associated proteins were changed within 12 hpt “Fig 5A–5D”, which further supported the differential-display proteins identified with the 2-D gel.

## 4. Discussion

To answer the immediate need to protect rice production by deploying durable resistance and novel strategies against various diseases, a variety of transgenic rice cultivars and pathogen elicitors have been developed [1,27–29]. Several PR plant defense proteins have been identified that are induced in response to pathogen attack and treatment with an elicitor protein [30]. Our results here suggest that the expression of defense-related proteins including PR proteins can be modulated in rice leaves by treatment with the elicitor protein MoHrip2.

### 4.1. ROS and PCD-Related Proteins

In our study ROS and PCD-related proteins such as germin like protein (GLP) (N5), senescence activated gene 12 (SAG12) (N6) were newly induced, and peptidyl-prolyl *cis-trans* isomerase (D1), protein disulfide isomerase (D2), and dnaK (D3) proteins were downregulated in MoHrip2-treated samples compared with the control. GLPs have superoxide dismutase (SOD) activity and protect plants from oxidative stresses induced by abiotic and biotic factors [31] by breaking down superoxide anions, thus generating H_2_O_2_, which acts as an early signaling molecule for downstream defense responses and a defense response molecule against invading pathogens [32]. After exogenous treatment with H_2_O_2_, rice seedlings accumulated proteins relating to redox homeostasis, which maintain the redox status of the plant cell by activating defense reactions or overcoming the deleterious effects of oxidative stresses, and some of these redox enzymes might be repressed to maintain the concentration of H_2_O_2_ [33,34]. In our study, the dnaK heat shock proteins and isomerases were down-regulated in elicitor-treated plants compared with the buffer controls, which might account for H_2_O_2_ accumulation in rice leaves treated with MoHrip2. Besides, isomerases and dnaK proteins regulate protein folding and prevent protein denaturation [35]. The dnaK-type chaperones also known as heat shock proteins that play an important role in refolding of proteins that are denatured during stresses [36].

### 4.2. Transcriptional and Signal Transduction-Related Proteins

The zinc finger protein (ZFP) (N2), associated with defense-related transcriptional factor, and tricin synthase 1 (N4), SP3D (N7), and farnesyl transferase (N9), that are linked with signal transduction pathways, were newly induced in treated plants but not in the controls. Tricin synthase 1 catalyzes the methylation of tricitin, an important flavonoid, and binds to *S*-adenosyl-l-methionine, important steps in the synthesis of SA, JA and other polyamines [37]. ZFPs are cold- and drought-responsive proteins in plants [38]. Overexpression of ZFP245 enhances the activity of ROS-related enzymes and rice seedling resistance to oxidative stress [39]. SP3D and farnesyl transferase proteins negatively regulate mitogen-activated protein kinase (MAPK) and the abscisic acid (ABA) pathway, respectively [16,40]. The MAPK pathway regulates the activity of various transcription factors, such as WRKY, and enzymes such as protein kinases, and ultimately regulates the induction of PR proteins [16]. The ABA pathway is mostly related to developmental processes and abiotic resistance in plants [41]. Exogenous application of ABA, however, can also enhance plant susceptibility to fungal and bacterial diseases, while a mutation that causes ABA deficiency induces an enhanced resistance in tomato and Arabidopsis [42,43]. ABA has also been confirmed to interact antagonistically with the salicylic acid (SA) pathway [44]. Recently, we identified some marker genes related to the SA and JA pathways that were induced in MoHrip2-treated rice seedling (Unpublished data), suggesting that MoHrip2 might induce disease resistance in rice via the SA- or JA-related pathway. Further confirmation is needed to prove that MoHrip2-induced resistance in rice is a consequence of these defense-related pathways.

### 4.3. PR Proteins

PR proteins are distinguishing entities for system acquired resistance (SAR) in plants [45]. In our study, thaumatin-like protein (TLP, PR5) (N10) and PR10 (U4) proteins were induced in treated rice seedlings but not in the control. PR5 and PR10 are biomarkers of resistance against abiotic and biotic stresses [46]. Overexpression of PR5 proteins in susceptible rice cultivar enhances disease resistance against the bacterium *Xanthomonas oryzae* pv. *oryzae* and the fungus *Rhizoctonia solani* [47,48]. PR5 and PR10 proteins are also up-regulated in rice leaves treated with *M*. *oryzae*, *X*. *oryzae*, chitosan, and fungal elicitor [13–15,47].

### 4.4. Photosynthesis and Energy-Related Proteins

Four additional proteins that were identified “Table 2” in MoHrip2-treated samples were associated with photosynthesis and energy-related proteins, including the large subunit of ribulose bisphosphate carboxylase (RuBisCO) (U1), magnesium binding proteins (U2 and U3) and ATP binding protein 4-coumaroyl-CoA synthase 5 (N8). ATP binding protein 4-coumaroyl-CoA synthase 5 is a nitrogen-responsive protein and also induced by wounding [49]. Several reports described the effect of nitrogen concentrations (low or high) on the proteome of rice roots and leaves [49,50], and several energy-related proteins, including 4-coumaroyl-CoA synthase 5, are induced in response to nitrogen levels [35].

The level of the crucial photosynthetic enzyme RuBisCO is reduced in rice leaves treated with *M*. *oryzae*, and *X*. *oryzae*, because pathogen infection degrades the chloroplast [51]. Changes in the concentration Mg^+2^, the central atom of chlorophyll, in chloroplasts regulate the function of photosynthetic enzymes [52]. In our study, RuBisCO and magnesium ion (Mg^+2^) binding proteins were induced in elicitor-treated rice seedlings and might be strengthening the resistance induced by MoHrip2 in rice leaves against *M*. *oryzae*.

### 4.5. Conclusion

Using a proteomics approach to elucidate the effects of MoHrip2 in triggering the rice defense system, we identified differentially expressed proteins in rice leaves at 24 h after treatment with the elicitor MoHrip2. MoHrip2 induced rice basal defense responses such as ROS, PCD and defense-related proteins. ROS serves as an early precursor during biotic and abiotic stresses, while triggering defense-related pathways downstream in plants. Moreover, the up-regulation of photosynthesis and energy-related proteins further supports MoHrip2-induced defense in rice, which might be helpful in stress relief. Studies are now needed to identify a host receptor for MoHrip2 in rice leaves to elucidate the mechanism of MoHrip2-induced resistance in rice.

## Acknowledgments

We thank Beth E. Hazen for carefully reviewing the manuscript and editing the English. This research was supported by the National Natural Science Foundation of China (Grant No. 31371984).
